# Finite Element Analysis of the Microwave Ablation Method for Enhanced Lung Cancer Treatment

**DOI:** 10.3390/cancers13143500

**Published:** 2021-07-13

**Authors:** Marija Radmilović-Radjenović, Martin Sabo, Marta Prnova, Lukaš Šoltes, Branislav Radjenović

**Affiliations:** 1 Institute of Physics, University of Belgrade, Pregrevica 118, 11080 Belgrade, Serbia; bradjeno@ipb.ac.rs; 2 Faculty of Informatics and Information Technologies, Slovak University of Technology in Bratislava, Ilkovicova 2, 84216 Bratislava, Slovakia; martin.sabo@stuba.sk (M.S.); marta.prnova@stuba.sk (M.P.); lukas.soltes@stuba.sk (L.Š.)

**Keywords:** microwave ablation, tissue, lung cancer, damage, dielectric properties

## Abstract

**Simple Summary:**

Microwave ablation is a promising modality for treating cancerous tumor cells in patients with localized lung cancer who are non-surgical candidates. Microwave ablation requires the control of the elevation of temperature, ensuring the destruction of cancer cells without damaging healthy tissue. Despite the unquestionable benefits, such as enlarged ablation zones and reduced procedure times, the respiratory movement of the lungs may affect the development and evolution of the necrotic tissue. Apart from the experimental methods, computer modeling has proven to be a powerful approach to improving the ablative treatment’s performance. This study aims to provide a step forward in patient safety by delivering optimal conditions necessary for microwave ablation to be as effective as possible for curing lung cancer with minimized invasiveness and collateral damage. The primary goal is to transfer the treatment plan based on simulation outputs into a reliable and safe microwave ablation procedure.

**Abstract:**

Knowledge of the frequency dependence of the dielectric properties of the lung tissues and temperature profiles are essential characteristics associated with the effective performance of microwave ablation. In microwave ablation, the electromagnetic wave propagates into the biological tissue, resulting in energy absorption and providing the destruction of cancer cells without damaging the healthy tissue. As a consequence of the respiratory movement of the lungs, however, the accurate prediction of the microwave ablation zone has become an exceptionally demanding task. For that purpose, numerical modeling remains a primordial tool for carrying out a parametric study, evaluating the importance of the inherent phenomena, and leading to better optimization of the medical procedure. This paper reports on simulation studies on the effect of the breathing process on power dissipation, temperature distribution, the fraction of damage, and the specific absorption rate during microwave ablation. The simulation results obtained from the relative permittivity and conductivity for inflated and deflated lungs are compared with those obtained regardless of respiration. It is shown that differences in the dielectric properties of inflated and deflated lungs significantly affect the time evolution of the temperature and its maximum value, the time, the fraction of damage, and the specific absorption rate. The fraction of damage determined from the degree of tissue injury reveals that the microwave ablation zone is significantly larger under dynamic physical parameters. At the end of expiration, the ablation lesion area is more concentrated around the tip and slot of the antenna, and the backward heating effect is smaller. The diffuse increase in temperature should reach a certain level to destroy cancer cells without damaging the surrounding tissue. The obtained results can be used as a guideline for determining the optimal conditions to improve the overall success of microwave ablation.

## 1. Introduction

Lung cancer, also known as lung carcinoma, is characterized by uncontrolled cell growth in the tissues of the lung, typically without any symptoms in its earliest stages [1,2,3,4,5,6,7,8]. Although the origin could be in any part of the lung, 90–95% of lung cancers arise from the epithelial cells that line the respiratory tract from the trachea to the bronchi into bronchioles and alveolar sacs [9,10]. The two key types of lung cancer, that grow and spread differently, are small-cell lung cancers (SCLC) and non-small-cell lung cancers (NSCLC) [11,12]. Lung cancer is recognized as the second most common cancer and the most common cause of death due to cancer. According to data from the literature, the lung cancer five-year survival rate of 18.6% is lower than other types of cancer, such as colorectal (64.5%), breast (89.6%), and prostate (98.2%). However, if cancer is detected at an early stage, the five-year survival rate for lung cancer increases to up to 56% [13,14,15,16,17]. Therefore, the main aims of cancer diagnosis and treatment procedures are to cure or prolong the patient’s life, ensuring the best possible quality of life for cancer survivors.

Depending on the type and stage of cancer, treatments usually include, but are not limited to, a combination of surgery, chemotherapy, targeted therapy, immunotherapy, and radiation therapy [18,19,20,21]. Microwave ablation (MWA) is a relatively new method for treating NSCLC to destroy tumor cells and create localized areas of tissue necrosis with minimal damage to the surrounding normal tissues [22,23,24,25,26]. Microwaves emitted from the antenna inserted inside the tumor mass create a small region of heat that destroys the lung cancer cells [27]. In some cases, two or more antennas can be used to achieve a larger ablation zone in a shorter time [28,29,30]. MWA offers some advantages over radiofrequency ablation (RFA), because microwaves are less susceptible to heat sinks, deeper penetration into low-conductivity tissue, and uniform MWA zones unaffected by convective heat loss [31,32].

Knowledge of the microwave dielectric properties of tissues plays a crucial role in the application of certain medical techniques, including MWA [33,34,35]. Generally, conductivity is defined as the free path length and speed of the electron inside the material, whereas permittivity is related to the molecule’s dipole moment per volume [36]. The physical property that controls microwave penetration in the tissue is permittivity, affecting the diffusion of microwaves in the tumoral tissue [37]. It has been reported that the permittivity of tumor tissue is 10–20% greater than that of normal tissue [38,39]. In the case of diseased lungs, the dielectric properties vary compared with healthy tissue. Thus, the lungs respond best to MWA due to marked differences in permittivity between tumors and the surrounding tissue.

The lungs are two spongy organs that take in oxygen during inhalation and release carbon dioxide during exhalation. During breathing, the amount of air in the lungs varies, leading to changes in their physical parameters. Experimental data indicate that the electrical properties of the lung tissue depend on the condition of the tissue. Furthermore, the conductivity and the relative permittivity decrease with increasing air filling [40]. Different conduction properties of the lungs greatly affect the temperature distribution in the MWA zone, making accurate prediction and control of the ablation areas very demanding [41,42]. Besides the dielectric properties of the tissue, the microwave frequencies also determine the depth of the tissue penetration by microwave [43,44].

In this paper, the effect of the MWA on the tumoral tissue has been studied by using the heat transfer module implemented in the COMSOL Multiphysics platform (Comsol Multiphysics 1986–2020) [45,46]. The focus was put on the interaction between the electromagnetic waves with the tissue to treat lung cancers. Simulation conditions include microwave antenna operating at frequencies between 915 MHz and 2.45 GHz, taking into account the frequency dependence of the permittivity and conductivity of the lungs. The influence of the variation of the permittivity and conductivity of the lungs during respiration on power dissipation, temperature distribution, the fraction of necrotic tissue, and the specific absorption rate (SAR) were analyzed. It was found that differences in the dielectric properties of inflated and deflated lungs strongly affect the time evolution of the temperature and its maximum value, the time dependence of the fraction of damage, and SAR. On the other hand, the total power dissipation density and the temperature distributions are similar regardless of respiration. In addition, a fraction of the necrotic tissue of the inflated lungs is similar to that obtained regardless of respiration. The obtained simulation results confirm that simulations can be very useful for predicting the temperature distributions and the tissue deformation during microwave ablation, and therefore may be incorporated into treatment planning.

## 2. Numerical Method and Simulation Conditions

Calculations were performed using the COMSOL Multiphysics simulation package based on a multi-component plasma fluid model [45]. The heat transfer module was employed to simulate the thermal field distribution of microwave ablation, optimizing the probe position and the power input for tissue damage conditions [46,47]. The electric field mode was calculated using numerical methods for computational electromagnetics, while the temperature field distribution was calculated using the bioheat transfer equation.

The propagation of electromagnetic waves in a coaxial cable is mathematically expressed via the following expressions [46]:

(1) $${E=e}_{r}\frac{C}{r}e^{j\left(\omega t-kz\right)},$$

(2) $${H=e}_{\varphi }\frac{C}{rZ}e^{j\left(\omega t-kz\right)},$$

(3) Pav=∫rinroutRe(12E×H*)2πrdr=ezπC2Zln(routrin),

where *z* is the direction of propagation, ω is the angular frequency, and *k* is the propagation constant. Cylindrical coordinates *r*, ϕ, and *z* are centered on the axis of the coaxial cable. **P***_av_* is the time-averaged power flow in the cable, *Z* is the wave impedance in the dielectric of the cable, while *r_in_* and *r_out_* are the dielectric’s inner and outer radii, respectively. In the tissue, the electric field has a finite axial component while the magnetic field is purely in the azimuthal direction, so the wave equation can be rewritten in the following form [46]:

(4) $$\nabla \times \left({\left(\varepsilon_{r}-\frac{j\sigma }{\omega \varepsilon_{0}}\right)}^{-1}\nabla \times H_{\varphi }\right)-\mu_{r}k_{0}^{2}H_{\varphi }=0,$$

where ε_0_ and ε*_r_* represent the vacuum dielectric constant and relative dielectric constant of the tissue, respectively, while σ is the tissue conductivity. The time-dependent heat transfer problem is described by the following equation [46]:

(5) $$\rho c\frac{\partial T}{\partial t}=\nabla \times \left(k\nabla T\right)+\rho_{b}W_{b}c_{b}\left(T_{b}-T\right)+Q_{ext}+Q_{m},$$

where *ρ*, *c*, *k*, and *T* are the density, the heat capacity, the thermal conductivity, and the temperature of the tissue, respectively, while *ρ_b_*, *c_b_*, and *T_b_* are the density, the heat capacity, and the temperature of the blood, respectively. *W_b_* is the blood perfusion rate and *t* is the time, whereas the heat source from metabolism, *Q_m_*, is neglected in our model. The external heat source *Q_ext_* is computed as the resistive heat generated by the electromagnetic field [46]:

(6) Qext=12Re[(σ−jωε)E⋅E*].

The degree of tissue injury is denoted by α and expressed by the Arrhenius equation [46,47]:

(7) $$\frac{d\alpha }{dt}=A\exp\left(-\frac{\Delta E}{RT}\right),$$

where *A* and Δ*E* represent the frequency factor and the activation energy for irreversible damage reaction, respectively. Both parameters depend on the type of tissue. The absolute temperature is denoted by T, and the gas constant is denoted by R. The fraction of necrotic tissue, θ*_d_*, can be determined from the degree of tissue injury according to [46,47]: The fraction of necrotic tissue, θ*_d_*, can be determined from the degree of tissue injury according to the following [46,47]:

(8) $$\theta_{d}=1-\exp\left(-\alpha \right).$$

**Figure 1:**
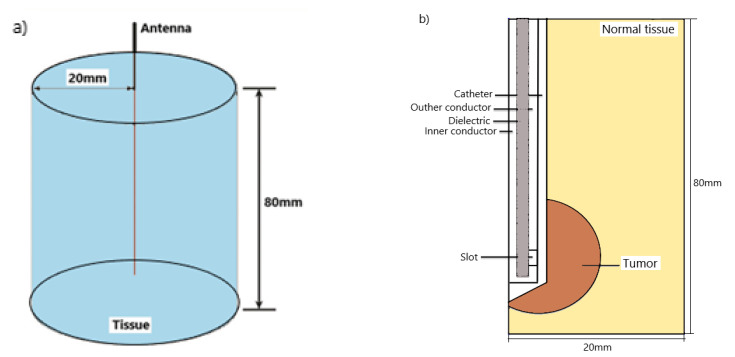
Schematic view of the (**a**) configuration of the antenna inserted into biological tissue used for microwave ablation therapy and (**b**) the microwave coaxial antenna enclosed in a catheter is composed of a dielectric, inner and outer conductors, and a slot. The tumor is assumed to have a spherical form.

**Figure 2:**
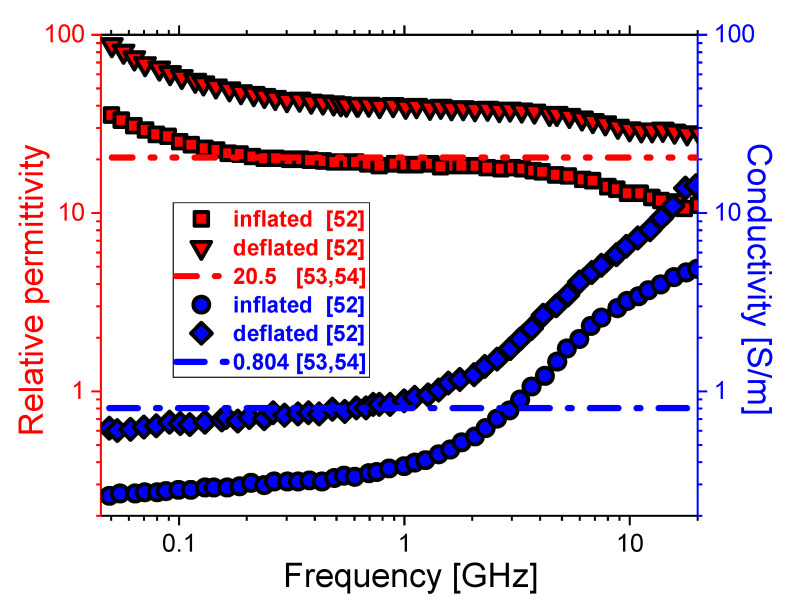
The frequency dependence of the relative permittivity (squares and down triangles) and conductivity (circles and diamonds) of the inflated and deflated lungs, respectively [52]. Curves correspond to the constant values of dielectric properties of the lungs regardless of respiration [53,54].

In MWA, a microwave coaxial antenna (MCA) is immersed in biological tissue, as illustrated in Figure 1a. The antenna geometry is composed of an inner conductor, a dielectric, and an outer conductor containing a ring-shaped slot, as shown in Figure 1b. For hygienic purposes, a plastic catheter surrounds the antenna [47,48,49,50]. Because of the axially symmetric domain of the microwave effect around the antenna, the cylinder model can be simplified to a semicircular model, which can be calculated with two-dimensional coordinates [47,48,49,50,51]. Calculations were carried out for the microwave frequency between 915 MHz and 2.45 GHz with an input power of 10 W. The frequency-dependent permittivity and conductivity corresponding to the inflated and deflated lungs are presented in Figure 2 [52]. With increasing frequency, the relative permittivity (squares and down triangles) decreases while the conductivity (circles and diamonds) increases. The conductivity varies slightly at frequencies lower than 1 GHz, while for higher frequencies, the rise is steeper.

Both the relative permittivity and conductivity during the deflation of the lungs are around two times larger than those during inflation. For comparison, values of 20.5 and 0.804 for permittivity and conductivity, respectively, are plotted by the curves [53,54]. Simulation parameters related to the lung properties include the average density of 385 kg/m^3^, the heat capacity of 3886 J/(kg.K), and thermal conductivity of 0.39 W/(m.K). The model assumes that the blood, with a density of 1060 kg/m^3^, enters the tissue at the body temperature *T*_b_ = 37 °C and is heated to a temperature of *T*. The blood’s specific heat capacity is 3639 J/(kg·K), while the blood perfusion rate is 0.0036 s^−1^ [47,50].

## 3. Results

**Figure 3:**
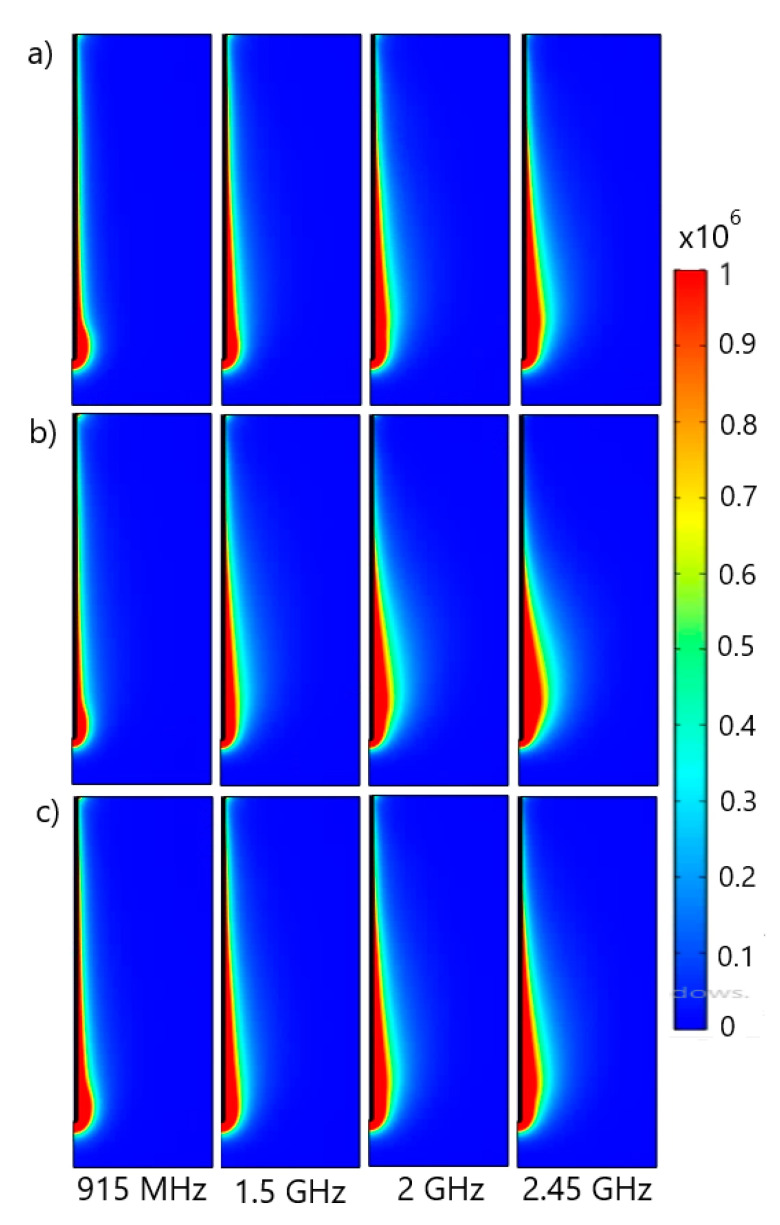
The total power dissipation density (expressed in W/m^3^) is calculated using the dielectric properties of (**a**) inflated lungs [52], (**b**) deflated lungs [52], and (**c**) constant permittivity and conductivity values [53,54] at microwave frequencies ranging from 915 MHz to 2.45 GHz.

When the tissue is exposed to the microwave field, the energy from the field is absorbed by the tissue, leading to the heating of the cancer cells. Figure 3 displays the microwave power density absorbed in (a) inflated lungs, (b) deflated lungs, and (c) the tissue regardless of respiration for the input power of 10 W at four different frequencies. MCA radiates energy throughout the biological tissue, and this energy is converted into heat that invades the tissues. Although the dielectric constants that characterize inflated and deflated lungs are different, the total power dissipation is similar. The absorbed power density is extremely high close to the antenna and decreases with distance. On the other hand, the extension of the heated zone slightly increases with the frequency. This extension occurs mainly in the vicinity of the tumor and has an oblong form. When average values of the lung’s permittivity and conductivity are accounted for, the shapes of the heated zone (Figure 3c) are similar to those corresponding to the inflated lungs (Figure 3a).

**Figure 4:**
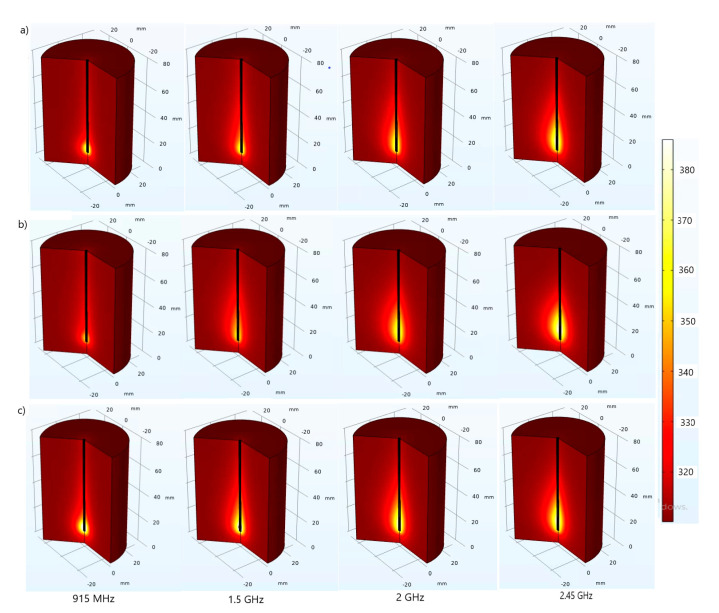
The 3D plot of the temperature (expressed in Kelvin) distributions obtained with the dielectric parameters that characterized (**a**) inflated lungs [52], (**b**) deflated lungs [52], and (**c**) constant values [53,54] at microwave frequencies between 915 MHz and 2.45 GHz.

**Figure 5:**
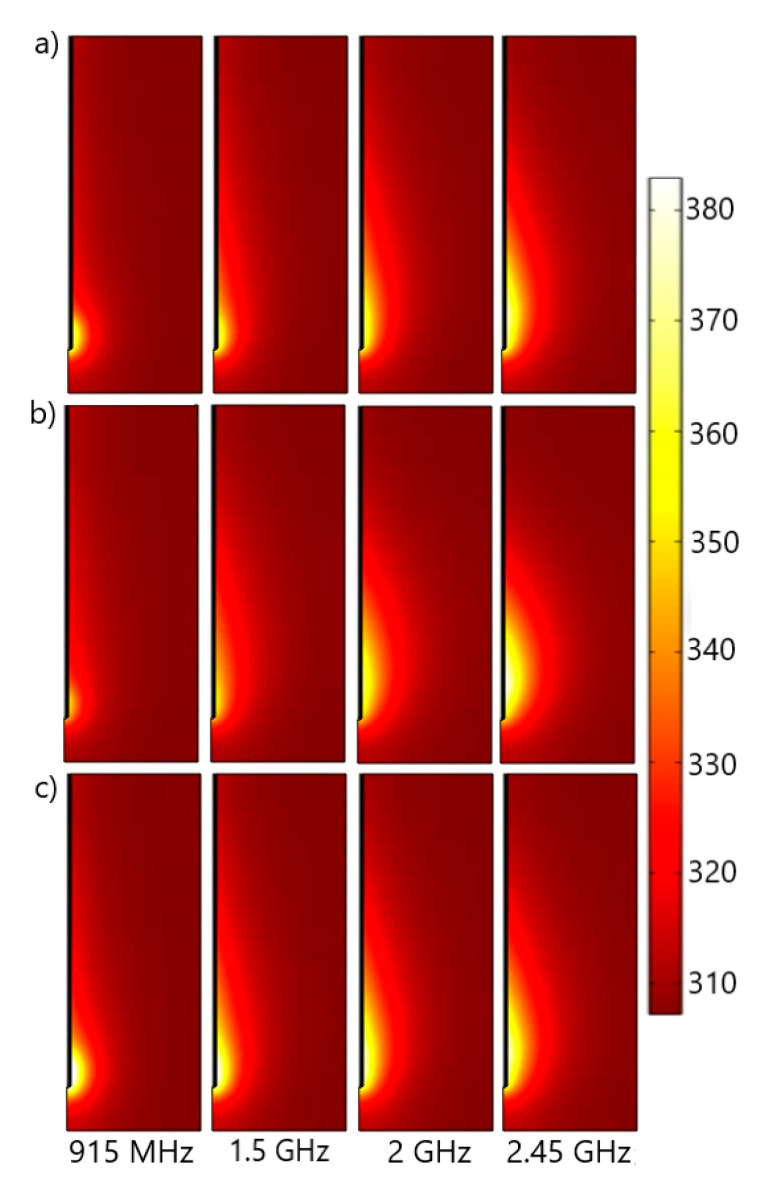
The temperature (expressed in Kelvin) distributions of the (**a**) inflated, (**b**) deflated lungs [52], and (**c**) tissues with constant dielectric properties [53,54] exposed to microwave frequencies from 915 MHz to 2.45 GHz.

Temperature profiles follow the heat-source distributions as observable from 3D (Figure 4) and 2D (Figure 5) plots, showing the steady-state temperature distributions in the lung tissue achieved after *t* = 600 s. The absorbed energy converted into thermal energy causes an increase in the tissue temperature. Whether respiration is taken into account or not, the temperature distribution is near ellipsoidal around the slot, reaching the highest values near the microwave antenna slot. As the distance from the antenna increases, the heat source becomes weaker, so the blood can maintain the tissue at a normal body temperature. It appears that the perfusion of blood limits the extent of the heated area.

**Figure 6:**
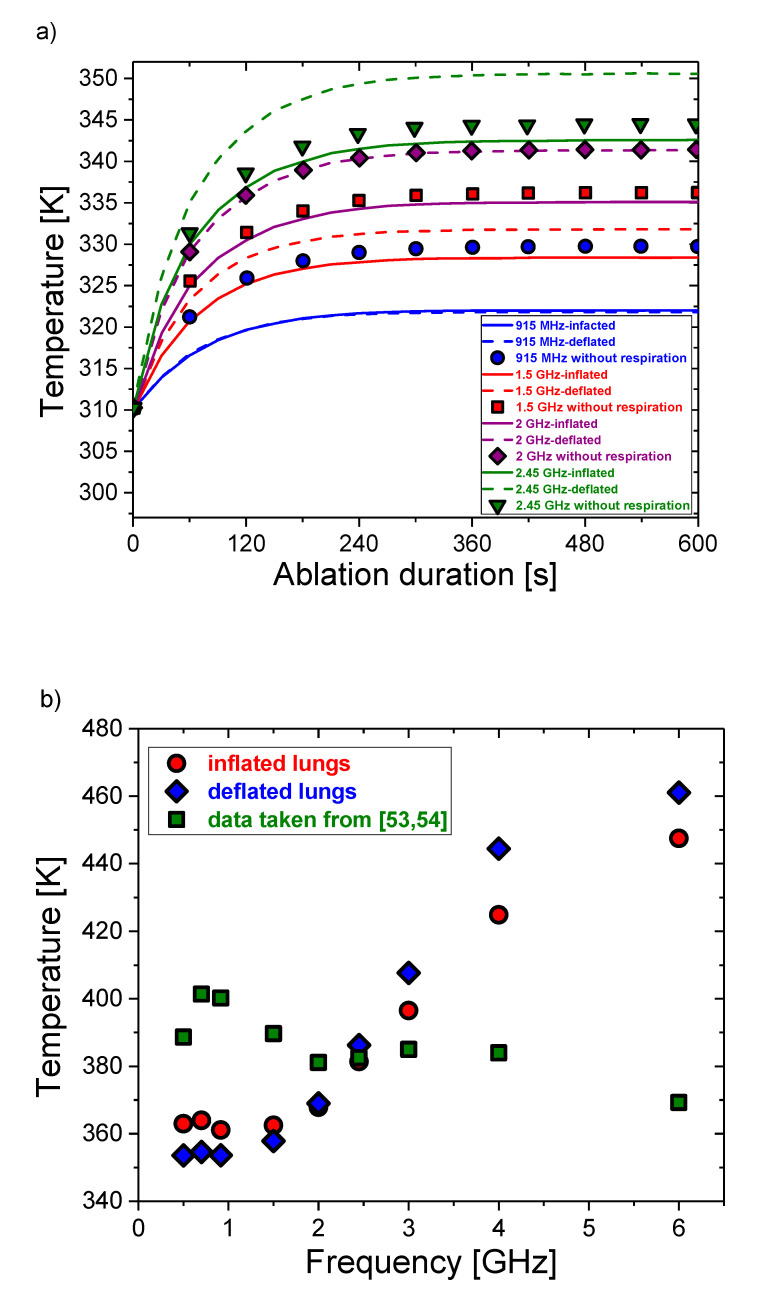
(**a**) Temporal evolution of the temperature. The results of calculations using permittivity and conductivity corresponding to inflation, deflation, and regardless of respiration are shown by solid curves, dash curves, and symbols, respectively. (**b**) The dependence of the maximum temperature on the frequency.

The temperature increase of the lungs during inflation (solid curve) and deflation (dash curve) is different, as shown in Figure 6a. In both cases, for the short duration of the ablation, the behavior of the curves is mainly governed by the source term. As a result, the temperature rises linearly. When a certain value is reached, the diffusion and heat conduction caused by blood perfusion become significant, resulting in a counterbalancing of the source term. Thus, a saturation regime takes place and the maximum temperature is reached. This temperature is reached about 30 s faster during deflation. Temperatures calculated regardless of respiration (symbols) have similar trends, but different values. The largest discrepancies are observed at 915 MHz and at 1.5 GHz. For 2 GHz and 2.45 GHz, the temperatures calculated with dielectric constants taken from [53,54] are similar to the values for the deflated and inflated lungs, respectively. The maximal temperatures during inflation (circles) and deflation (diamonds) as a function of frequency are depicted in Figure 6b. Although the trends are similar, the higher maximum temperature corresponds to a deflation above 915 MHz. Completely different trends are noticed when the effect of respiration on dielectric parameters is not considered (squares). Only at 2.45 GHz, the maximum temperature calculated regardless of respiration agrees with the values related to respiration. At higher frequencies, these maximum temperatures are significantly lower than those calculated for the inflated and deflated lungs. All of the plotted curves can be very useful for determining the optimal value of the treatment time.

**Figure 7:**
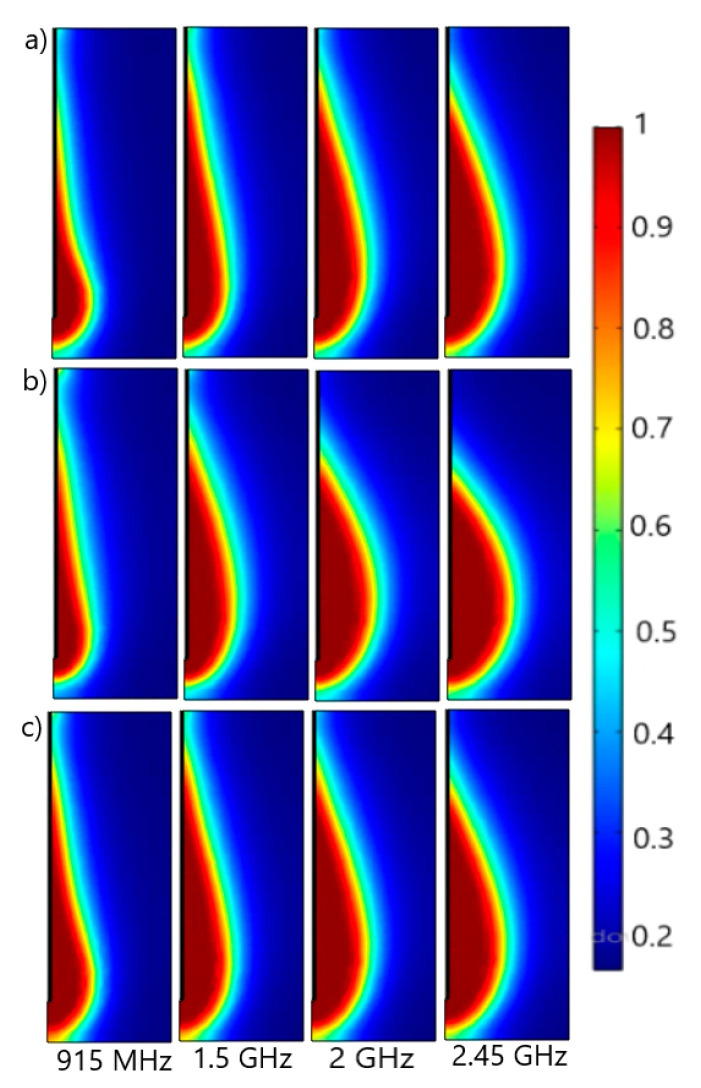
Fraction of necrotic tissue obtained with dielectric constants for the (**a**) inflated lungs [52], (**b**) deflated lungs [52], and (**c**) constant values [53,54], after 600 s of exposure to a frequency between 915 MHz and 2.45 GHz.

Figure 7 contains a numerical prediction of the fraction of necrotic tissue after 600 s of exposure to frequencies between 915 MHz and 2.45 GHz. Whether the lungs are inflated (Figure 7a) or deflated (Figure 7b), or respiration is not taken into account (Figure 7c), the damage zones are concentrated around the tip and slot of the antenna, while the backward heating effect is smaller. The extension of the necrotic tissue increases with the frequency. Although these extensions take place mainly in the vicinity of the tumor, they are more oblong in the case of inflated lungs (Figure 7a) and when using dielectric constants regardless of respiration (Figure 7c). For deflated lungs (Figure 7b), increasing the frequency leads to changes in the necrotic tissue’s shape from oblong to more rounded.

**Figure 8:**
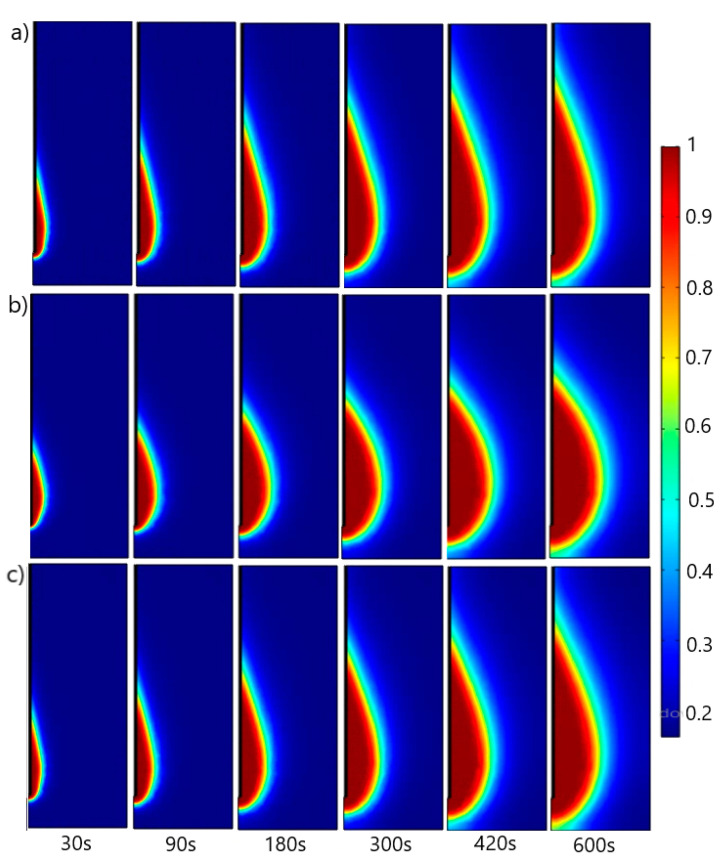
The time evolution of the fraction of damage of the tissue exposed to 2.45 GHz. Calculations were performed using dielectric constants for (**a**) inflated lungs [52], (**b**) deflated lungs [52], and (**c**) values taken from [53,54].

The time evolution of the necrotic tissue exposed to a microwave frequency of 2.45 GHz is shown in Figure 8. First, the tumor damage gradually increases and then reaches a saturation region, which presents the completion time of tumor necrosis. Because of the variation in dielectric properties of the lungs during respiration, the necrotic tissue has an oblong form (Figure 8a) for inflated lungs and a rounded form for deflated lungs (Figure 8b). Regardless of inflation or deflation, the oblong form of the necrotic tissue (Figure 8c) is similar to that corresponding to the inflated lungs (Figure 8a).

**Figure 9:**
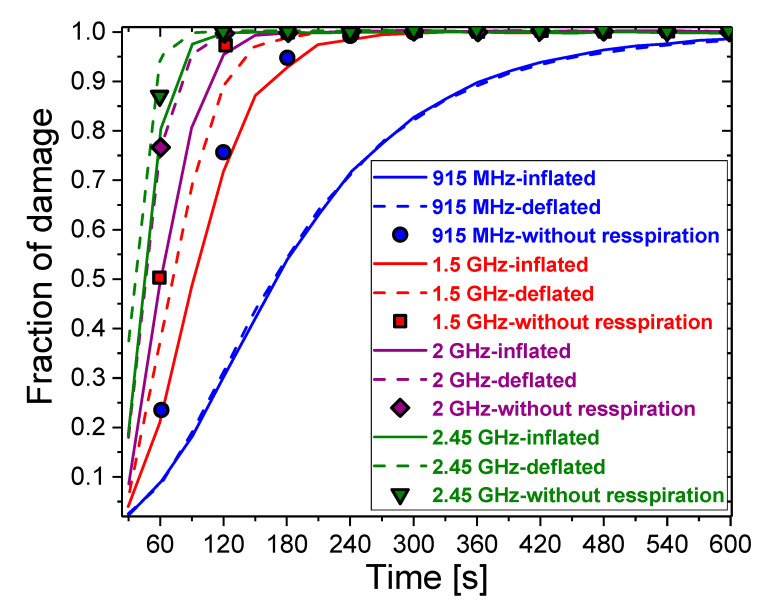
The necrotic tissue during ablation time for inflation (solid curves), deflation (dash curves), and regardless of respiration (symbols) of the lungs, exposed to microwave frequencies between 915 MHz and 2.45 GHz.

As can be seen from the temporal evolution of the necrotic tissue (Figure 9), the fraction of damage increases with increasing time. With or without the respiration effect on the dielectric constants, all of the curves have similar trends with a difference in magnitude. The time for reaching the maximal fraction is shorter during inflation of the lungs. The shortest time required for the fraction to become 1 is obtained when deflated lungs are exposed to 2.45 GHz. On the other hand, for 915 MHz, the time for reaching the maximum fraction of damage for the inflated and deflated lungs is almost the same. The results for inflated and deflated lungs disagree with those obtained by not taking into account respiration (symbols), especially for 915 MHz. For this frequency, the fraction of damage to inflated lungs is less than 5% higher than that for deflated lungs. When respiration is not considered, however, the fraction of damage increases by up to 78%. As the frequency increases, the difference between them decreases. At 2 GHz, the results obtained with constant values from [53,54] (diamonds) agree with the results for deflated lungs. For 2.45 GHz, however, they (down triangles) better agree with the results for inflated lungs. As the results were obtained for the input power of 10 W, one may conclude that the ablation of tumor cells can be achieved over a long period without damaging healthy cells by using a small input power.

**Figure 10:**
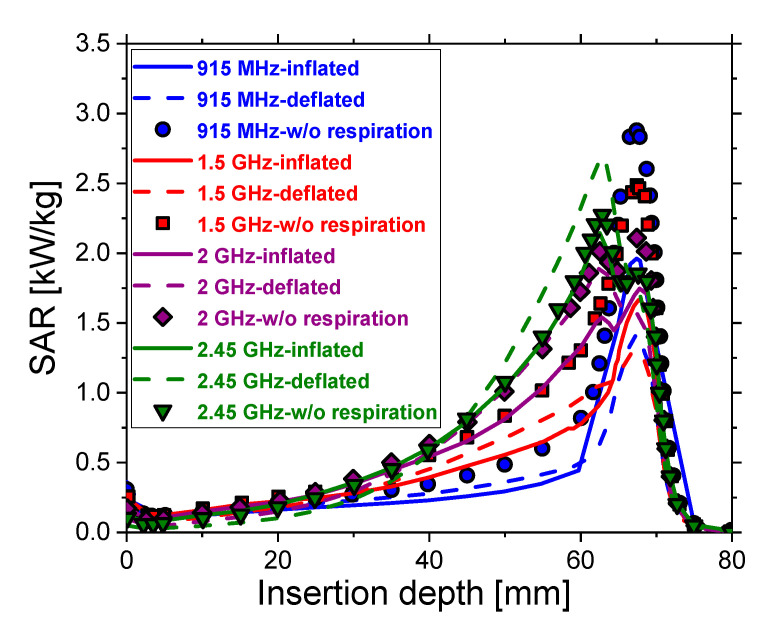
Axial profile of the specific absorption rate (SAR) during inflation (solid curves), deflation (dash curves), and regardless of respiration (symbols) at microwave frequencies in the range of 915 MHz to 2.45 GHz.

The specific absorption rate (SAR), defined as the ratio of absorbed heat power and tissue density, is shown in Figure 10. Regardless of respiration, for all frequencies, SAR increases slowly along the axis parallel to the antenna, reaches a peak around the slot of the antenna, and then decreases. The maximal values of SAR correspond to the tumor region. For frequencies of 915 MHz and 1.5 GHz, the maximal values are obtained for dielectric properties regardless of respiration (symbols), while those for inflated (solid curves) and deflated lungs (dash curves) are significantly lower. For 2 GHz, values for inflated lungs (purple solid curve) are the lowest, while SAR values for deflated lungs (purple dash curve) and without respiration (purple diamonds) are similar. Finally, at 2.45 GHz, SAR values for deflated lungs are the highest. For 915 MHz (blue), 1.5 GHz (red), 2 GHz (purple), and 2.45 GHz (olive), differences in the maximal SAR values related to the inflated and deflated lungs are around 28%, 22%, 8.5%, and 20%, respectively. Differences between the maximal values of SAR associated with inflated lungs and regardless of respiration are 32%, 33%, 13%, and 5%, respectively. On the other hand, maximal SAR values regardless of respiration differ from those of deflated lungs by about 51%, 48%, 5%, and 15%, respectively.

## 4. Conclusions

This paper reports on simulation studies of the influence of the respiratory movement of the lungs on the effect of microwave ablation on the tumoral tissues. Calculations were performed using the heat transfer module implemented in the COMSOL Multiphysics platform [45,46]. Simulation conditions encompass microwave antenna operating at a frequency range from 915 MHz to 2.45 GHz, both with and without frequency dependence on the permittivity and conductivity of the lungs [52,53]. The analysis of the obtained simulation results reveals that regardless of respiration, the total power dissipations are large close to the antenna while decreasing with the distance. The frequency affects the extension of the oblong heated zone, mainly in the vicinity of the tumor. One of the main characteristics related to the effective performance of MWA is the temperature profile, which follows the heat-source distribution. With the increased ablation time, the temperature increases, and upon saturation, diffusion and heat conduction due to blood perfusion become significant. The difference between the maximum temperatures associated with inflation and deflation is up to 19 K. Without respiration, the maximum temperatures are lower, about 78 K and 92 K, than those corresponding to the inflated and deflated lungs, respectively. Whether the lungs are inflated or deflated, the damage zones are concentrated around the tip and slot of the antenna, while the backward heating effect is smaller. For all frequencies, the extension of the necrotic tissue occurs mainly in the vicinity of the tumor. For inflated lungs, such a zone is more oblong compared with the rounded one for deflated lungs. The fraction of damage to inflated lungs is lower than that of the deflated lungs. Although it seems that the dielectric properties of the lungs do not change the necrotic tissue significantly, even small changes are important for completing the time of tumor necrosis and contributing to the preservation of healthy tissue. Regardless of the respiratory movement of the lungs, the time required for complete necrosis is the shortest at 2.45 GHz. The maximal SAR values are achieved within the tumor region. Based on all of the above, one may conclude that the breathing process plays a significant role in the treatment of lung cancer by using the microwave ablation modality. The obtained simulation results may serve as an essential basis for the improvement of the microwave ablation procedure to destroy the maximal part of the tumor while conserving the healthy tissue. However, it has to be pointed out that before any clinical application of presented results, histological assessment is mandatory.

## Data Availability

The data are available from the corresponding authors upon reasonable request.
